# Supplementary material for: Dissecting the economic impact of soybean diseases in the United States over two decades
Source: PLoS One. 2020 Apr 2;15(4):e0231141. doi: 10.1371/journal.pone.0231141 (PMC7117771; doi:10.1371/journal.pone.0231141)
Supplement: S1 Table — (DOCX) [file pone.0231141.s001.docx]

**Supplementary table 1**. Common names of the diseases considered in the study and Latin binomial of the pathogen(s) causing each disease.

| **Disease common name** | **Pathogen/s causing the disease** |
| --- | --- |
| Anthracnose | *Colletotrichum truncatum* and several related species |
| Bacterial diseases | *Pseudomonas savastanoi* pv. *glycines*; *P. syringae* pv. *tabaci*; |
|  | *Xanthomonas axonopodis* pv. *glycines*; others |
| Brown stem rot | *Phialophora gregata* |
| Cercospora leaf blight and/or purple seed stain | *Cercospora flagellaris*, *C. kikuchii*, *C. sigesbeckiae* |
| Charcoal rot | *Macrophomina phaseolina* |
| Downy mildew | *Peronospora manshurica* |
| Frogeye leaf spot | *Cercospora sojina* |
| Fusarium wilt and root rot | *Fusarium* spp*.* |
| Other diseases | See footnote^a^ |
| Diaporthe-Phomopsis | *Diaporthe longicolla* |
| Phytophthora root and stem rot | *Phytophthora sojae* |
| Pod and stem blight | *Diaporthe sojae* |
| Rhizoctonia aerial blight | *Rhizoctonia solani* |
| Root-knot and other nematodes | See footnote^b^ |
| Sclerotinia stem rot (White mold) | *Sclerotinia sclerotiorum* |
| Seedling diseases | See footnote^c^ |
| Septoria brown spot | *Septoria glycines* |
| Southern blight | *Sclerotium rolfsii* |
| Soybean cyst nematode | *Heterodera glycines* |
| Soybean rust | *Phakopsora pachyrhizi* |
| Stem canker | *Diaporthe aspalathi*; *D. caulivora* |
| Sudden death syndrome | *Fusarium virguliforme* |
| Viruse diseases | See footnote^d^ |

^a^ Includes: black root rot, Cercospora leaf blight, *Cylindrocladium parasticum* (red crown rot), green stem syndrome, Neocosmospora root rot, Pythium root rot, target spot, and Texas root rot.

^b^ Includes: *Rotylenchulus reniformis* (reniform nematode), *Belonolaimus longicaudatus* (sting nematode), and *Meloidogyne* (root-knot nematodes), *Helicotylenchus* (spiral nematodes), *Hoplolaimus* (lance nematodes), *Paratrichodorus* (stubby root nematodes), and *Pratylenchus* spp. (lesion nematodes).

^c^ Includes: seedling diseases caused by a complex of organisms such as multiple species of *Fusarium*, *Pythium*, *Phomopsis*, and/or *Rhizoctonia solani*.

^d^ Includes: *Alfalfa mosaic virus*, *Bean pod mottle virus*, *Bean yellow mosaic virus*, *Peanut mottle virus*, *Soybean dwarf virus*, *Soybean mosaic virus*, *Soybean vein necrosis virus*, *Tobacco ringspot virus*, *Tobacco streak virus*, and *Tomato spotted wilt virus*.
