# Supplementary material for: Dissecting the economic impact of soybean diseases in the United States over two decades
Source: PLoS One. 2020 Apr 2;15(4):e0231141. doi: 10.1371/journal.pone.0231141 (PMC7117771; doi:10.1371/journal.pone.0231141)
Supplement: S2 Table — (DOCX) [file pone.0231141.s002.docx]

**Supplementary table 2.** Total state-wide economic losses due to soybean diseases (in million USD) from 12 northern states in the United States from 1996 to 2016.

|  | **State (northern United States)** | | | | | | | | | | | |  |
| --- | --- | --- | --- | --- | --- | --- | --- | --- | --- | --- | --- | --- | --- |
| **Year** | **IA** | **IL** | **IN** | **KS** | **MI** | **MN** | **ND** | **NE** | **OH** | **PA** | **SD** | **WI** | **Total** |
| 1996 | 2,999.76 | 439.61 | 344.94 | 33.08 | 90.71 | 169.27 | 11.98 | 83.50 | 421.73 | 24.23 | 79.34 | 19.51 | **4,717.66** |
| 1997 | 2,154.95 | 385.80 | 351.59 | 81.40 | 78.95 | 494.00 | 14.69 | 97.72 | 469.19 | 4.36 | 139.06 | 49.10 | **4,320.81** |
| 1998 | 2,834.12 | 545.82 | 310.28 | 331.05 | 77.24 | 76.89 | 10.78 | 69.66 | 118.43 | 3.69 | 113.60 | 26.86 | **4,518.43** |
| 1999 | 2,015.69 | 342.85 | 230.16 | 136.93 | 62.52 | 159.08 | 16.21 | 64.62 | 51.19 | 0.71 | 76.21 | 41.59 | **3,197.76** |
| 2000 | 692.16 | 905.79 | 262.38 | 124.09 | 55.54 | 159.76 | 35.56 | 101.68 | 264.50 | 10.82 | 90.69 | 59.35 | **2,762.32** |
| 2001 | 721.81 | 287.83 | 213.65 | 35.64 | 18.95 | 255.54 | 26.96 | 54.32 | 187.45 | 4.28 | 71.85 | 58.47 | **1,936.75** |
| 2002 | 607.00 | 633.82 | 191.55 | 105.53 | 55.16 | 394.40 | 34.35 | 84.04 | 194.97 | 85.14 | 86.82 | 72.20 | **2,544.98** |
| 2003 | 860.41 | 1,217.22 | 243.45 | 275.29 | 41.95 | 119.73 | 29.18 | 48.46 | 493.88 | 20.45 | 103.43 | 82.29 | **3,535.73** |
| 2004 | 1,104.48 | 853.99 | 240.01 | 49.17 | 26.40 | 380.64 | 28.64 | 36.70 | 316.11 | 13.59 | 68.28 | 83.37 | **3,201.38** |
| 2005 | 201.99 | 441.68 | 221.43 | 92.39 | 40.50 | 239.31 | 39.03 | 36.02 | 252.18 | 28.50 | 72.45 | 103.89 | **1,769.37** |
| 2006 | 439.69 | 701.11 | 282.85 | 149.54 | 43.83 | 526.61 | 56.54 | 38.22 | 504.30 | 14.44 | 91.83 | 141.88 | **2,990.85** |
| 2007 | 361.41 | 1,143.90 | 299.79 | 70.16 | 45.04 | 584.36 | 97.32 | 37.89 | 254.92 | 36.35 | 213.74 | 166.87 | **3,311.76** |
| 2008 | 426.92 | 803.43 | 446.95 | 172.74 | 166.91 | 524.18 | 65.02 | 47.07 | 758.49 | 25.78 | 276.63 | 242.44 | **3,956.55** |
| 2009 | 653.80 | 1,086.33 | 383.56 | 292.28 | 44.29 | 558.17 | 142.98 | 49.93 | 761.88 | 17.71 | 245.52 | 510.16 | **4,746.62** |
| 2010 | 947.93 | 1,151.28 | 330.12 | 342.77 | 442.34 | 919.81 | 236.23 | 62.39 | 379.24 | 25.98 | 432.10 | 464.39 | **5,734.59** |
| 2011 | 340.48 | 622.12 | 187.67 | 194.09 | 1,520.28 | 666.20 | 312.24 | 154.47 | 325.45 | 104.87 | 333.74 | 355.04 | **5,116.64** |
| 2012 | 735.39 | 434.92 | 179.56 | 464.33 | 742.89 | 553.13 | 138.17 | 151.78 | 335.75 | 110.67 | 418.49 | 1,277.32 | **5,542.41** |
| 2013 | 344.66 | 564.86 | 192.61 | 236.08 | 307.43 | 591.09 | 141.44 | 115.89 | 1,576.64 | 72.51 | 470.17 | 278.50 | **4,891.90** |
| 2014 | 504.08 | 585.19 | 391.17 | 170.50 | 375.90 | 550.36 | 145.81 | 118.69 | 824.75 | 74.63 | 319.86 | 307.86 | **4,368.80** |
| 2015 | 493.66 | 822.14 | 247.67 | 49.52 | 284.15 | 469.69 | 123.48 | 103.91 | 1,327.47 | 71.81 | 213.71 | 178.42 | **4,385.62** |
| 2016 | 439.35 | 704.81 | 277.76 | 80.48 | 198.41 | 436.07 | 181.46 | 87.89 | 422.07 | 56.67 | 217.33 | 239.26 | **3,341.55** |
| **Total** | **19,879.72** | **14,674.52** | **5,829.16** | **3,487.07** | **4,719.38** | **8,828.29** | **1,888.05** | **1,644.86** | **10,240.59** | **807.19** | **4,134.87** | **4,758.78** | **80,892.48** |
