# Supplementary material for: Dissecting the economic impact of soybean diseases in the United States over two decades
Source: PLoS One. 2020 Apr 2;15(4):e0231141. doi: 10.1371/journal.pone.0231141 (PMC7117771; doi:10.1371/journal.pone.0231141)
Supplement: S3 Table — (DOCX) [file pone.0231141.s003.docx]

**Supplementary table 3.** Total state-wide economic losses due to soybean diseases (in million USD) from 16 southern states in the United States from 1996 to 2016.

|  |  | | **State (southern United States)** | | | | | | | | | | | | | | |  |
| --- | --- | --- | --- | --- | --- | --- | --- | --- | --- | --- | --- | --- | --- | --- | --- | --- | --- | --- |
| **Year** | **AL** | **AR** | | **DE** | **FL** | **GA** | **KY** | **LA** | **MD** | **MO** | **MS** | **NC** | **OK** | **SC** | **TN** | **TX** | **VA** | **Total** |
| 1996 | 8.07 | 135.10 | | 2.06 | 1.57 | 12.12 | 43.70 | 64.30 | 4.60 | 148.55 | 93.11 | 36.23 | 5.30 | 29.82 | 25.53 | 13.66 | 2.36 | **626.07** |
| 1997 | 11.80 | 121.93 | | 3.19 | 1.77 | 7.53 | 43.31 | 81.35 | 5.72 | 200.30 | 105.87 | 50.84 | 7.15 | 21.31 | 30.59 | 16.02 | 3.04 | **711.70** |
| 1998 | 8.18 | 74.55 | | 2.86 | 0.47 | 5.52 | 42.59 | 41.13 | 3.43 | 125.78 | 246.32 | 24.95 | 5.85 | 16.12 | 53.37 | 9.32 | 3.32 | **663.74** |
| 1999 | 4.42 | 84.87 | | 3.61 | 0.36 | 2.57 | 24.97 | 35.59 | 8.87 | 89.25 | 97.11 | 24.74 | 6.91 | 14.38 | 13.99 | 5.69 | 3.19 | **420.51** |
| 2000 | 1.25 | 79.54 | | 0.19 | 0.13 | 3.36 | 31.32 | 29.13 | 5.21 | 86.89 | 34.22 | 50.85 | 2.77 | 7.92 | 25.51 | 6.36 | 5.90 | **370.55** |
| 2001 | 2.66 | 87.67 | | 3.26 | 0.17 | 2.07 | 30.48 | 14.11 | 2.97 | 64.55 | 93.48 | 38.52 | 3.16 | 12.34 | 125.21 | 1.58 | 4.40 | **486.62** |
| 2002 | 1.90 | 118.51 | | 1.93 | . | 2.85 | 31.21 | 36.19 | 3.08 | 64.71 | 101.17 | 44.50 | 2.16 | 11.70 | 154.89 | 9.59 | 2.84 | **587.24** |
| 2003 | 5.06 | 118.78 | | 2.79 | . | 7.22 | 30.86 | 48.00 | 5.17 | 78.57 | 90.00 | 79.64 | 2.84 | 22.10 | 178.85 | 7.64 | 10.51 | **688.04** |
| 2004 | 6.50 | 134.33 | | 1.72 | 1.02 | 6.43 | 21.33 | 53.04 | 5.09 | 63.71 | 138.62 | 78.38 | 2.89 | 26.55 | 163.63 | 12.49 | 15.14 | **730.87** |
| 2005 | 3.33 | 42.25 | | 2.14 | 0.15 | 5.96 | 24.06 | 19.95 | 3.47 | 49.74 | 60.38 | 39.30 | 4.95 | 9.74 | 6.25 | 8.27 | 13.57 | **293.50** |
| 2006 | 17.40 | 53.65 | | 0.99 | 0.06 | 4.26 | 24.13 | 22.11 | 2.73 | 67.54 | 39.48 | 39.31 | 3.97 | 10.85 | 113.38 | 6.48 | 10.62 | **416.97** |
| 2007 | 4.37 | 74.84 | | 1.12 | 0.80 | 10.07 | 35.31 | 23.61 | 2.26 | 70.48 | 25.70 | 23.71 | 5.76 | 7.98 | 41.27 | 2.22 | 13.24 | **342.74** |
| 2008 | 17.08 | 69.51 | | 1.88 | 4.55 | 19.80 | 35.72 | 51.64 | 2.80 | 119.76 | 47.20 | 59.93 | 8.27 | 16.88 | 110.64 | 0.77 | 15.82 | **582.26** |
| 2009 | 21.79 | 107.21 | | 2.81 | 2.32 | 36.81 | 41.49 | 63.89 | 1.53 | 133.46 | 121.01 | 58.17 | 11.26 | 17.33 | 405.37 | 0.94 | 17.93 | **1,043.31** |
| 2010 | 10.72 | 91.15 | | 3.93 | 0.40 | 11.39 | 31.83 | 27.87 | 3.51 | 158.89 | 145.10 | 47.18 | 10.01 | 13.07 | 129.27 | 0.41 | 18.46 | **703.21** |
| 2011 | 9.32 | 131.53 | | 9.14 | 0.17 | 3.58 | 37.98 | 26.64 | 4.11 | 138.91 | 83.99 | 32.05 | 3.32 | 9.79 | 97.76 | 0.00 | 23.81 | **612.10** |
| 2012 | 14.99 | 179.60 | | 8.09 | 0.67 | 11.36 | 70.44 | 67.65 | 6.02 | 76.40 | 103.84 | 58.50 | 4.99 | 18.40 | 140.35 | 0.37 | 30.98 | **792.66** |
| 2013 | 19.39 | 204.51 | | 9.77 | 1.23 | 5.72 | 96.56 | 59.15 | 27.75 | 485.22 | 141.35 | 46.73 | 8.76 | 8.45 | 172.91 | 0.09 | 29.63 | **1,317.23** |
| 2014 | 13.59 | 184.98 | | 3.48 | 0.71 | 9.91 | 104.41 | 161.44 | 8.07 | 265.32 | 319.11 | 81.57 | 7.31 | 14.39 | 127.13 | 0.32 | 29.46 | **1,331.20** |
| 2015 | 15.77 | 159.99 | | 2.92 | 0.22 | 7.31 | 70.92 | 91.77 | 7.54 | 197.47 | 170.42 | 51.10 | 6.77 | 13.38 | 102.16 | 0.10 | 23.15 | **920.99** |
| 2016 | 8.71 | 163.27 | | 0.98 | 0.72 | 8.89 | 73.72 | 121.25 | 4.87 | 137.44 | 191.96 | 49.23 | 8.34 | 12.93 | 142.04 | 0.35 | 22.04 | **946.74** |
| **Total** | **206.31** | **2,417.77** | | **68.85** | **17.49** | **184.72** | **946.34** | **1,139.81** | **118.79** | **2,822.92** | **2,449.44** | **1,015.44** | **122.73** | **315.43** | **2,360.11** | **102.68** | **299.41** | **14,588.24** |
