# Supplementary material for: Dissecting the economic impact of soybean diseases in the United States over two decades
Source: PLoS One. 2020 Apr 2;15(4):e0231141. doi: 10.1371/journal.pone.0231141 (PMC7117771; doi:10.1371/journal.pone.0231141)
Supplement: S4 Table — (DOCX) [file pone.0231141.s004.docx]

**Supplementary table 4.** Estimated annual (from 1996 to 2016) soybean economic losses (in million USD) due to diseases across 28 states within the United States (AL, AR, DE, FL, GA, IA, IL, IN, KS, KY, LA, MD, MI, MN, MO, NC, ND, NE, OH, OK, PA, SC, SD, TN, TX, VA,and WI).

|  | Year^a^ | | | | | | | | | | | | | | | | | | | | | |  | |
| --- | --- | --- | --- | --- | --- | --- | --- | --- | --- | --- | --- | --- | --- | --- | --- | --- | --- | --- | --- | --- | --- | --- | --- | --- |
| **Disease^b^** | **1996** | **1997** | **1998** | **1999** | **2000** | **2001** | **2002** | **2003** | **2004** | **2005** | **2006** | **2007** | **2008** | **2009** | **2010** | **2011** | **2012** | **2013** | **2014** | **2015** | **2016** | **Total** | |  |
| ANT | 46.2 | 102.5 | 58.3 | 124.3 | 41.4 | 54.3 | 46.6 | 117.8 | 132.1 | 106.8 | 155.0 | 128.0 | 134.2 | 220.8 | 107.3 | 85.6 | 52.8 | 41.1 | 38.1 | 57.4 | 73.5 | **1,924** | |  |
| BAC | 9.0 | 9.3 | 7.6 | 3.2 | 2.7 | 2.1 | 5.1 | 3.4 | 5.4 | 8.0 | 30.9 | 77.2 | 73.8 | 49.2 | 87.7 | 101.4 | 35.1 | 12.3 | 22.3 | 26.8 | 63.4 | **636** | |  |
| BSR | 461.8 | 265.2 | 116.3 | 73.6 | 78.2 | 74.4 | 56.7 | 129.7 | 119.7 | 53.8 | 147.0 | 143.5 | 149.5 | 239.1 | 183.3 | 232.5 | 248.0 | 154.0 | 154.4 | 171.4 | 147.5 | **3,399** | |  |
| CLBPSS | 119.1 | 39.9 | 38.2 | 43.3 | 13.2 | 27.1 | 60.1 | 45.5 | 33.2 | 31.7 | 25.6 | 31.6 | 87.7 | 158.4 | 92.7 | 71.4 | 71.5 | 74.2 | 121.1 | 439.7 | 161.5 | **1,787** | |  |
| CHAR | 142.5 | 179.1 | 412.2 | 316.3 | 217.3 | 114.9 | 270.4 | 916.4 | 85.3 | 174.5 | 210.0 | 362.1 | 261.6 | 102.7 | 515.0 | 607.0 | 1,361.2 | 597.6 | 305.3 | 220.0 | 113.0 | **7,484** | |  |
| DIAPHOM | 40.1 | 16.2 | 75.8 | 31.8 | 58.7 | 69.1 | 131.1 | 42.6 | 90.8 | 45.8 | 37.1 | 24.4 | 70.3 | 288.0 | 87.0 | 61.2 | 80.3 | 87.6 | 115.8 | 50.6 | 67.5 | **1,572** | |  |
| DM | 3.8 | 2.6 | 6.8 | 0.9 | 4.5 | 0.7 | 0.6 | 3.1 | 4.0 | 4.3 | 43.9 | 17.2 | 36.3 | 72.8 | 124.4 | 70.9 | 13.7 | 17.0 | 14.8 | 44.3 | 24.8 | **512** | |  |
| FLS | 10.9 | 13.7 | 31.5 | 9.9 | 29.0 | 18.8 | 61.4 | 102.7 | 95.8 | 33.9 | 108.8 | 120.8 | 81.2 | 99.8 | 163.5 | 82.7 | 61.4 | 258.0 | 216.8 | 172.9 | 187.4 | **1,961** | |  |
| FW | 13.6 | 24.3 | 23.8 | 25.2 | 9.9 | 59.0 | 122.1 | 63.9 | 70.6 | 40.2 | 47.7 | 70.1 | 129.6 | 97.3 | 140.1 | 289.4 | 302.6 | 130.2 | 47.3 | 31.1 | 40.5 | **1,778** | |  |
| OTHER^c^ | 20.1 | 121.3 | 10.2 | 13.7 | 26.7 | 17.3 | 18.7 | 25.1 | 76.7 | 4.0 | 17.0 | 1.9 | 9.0 | 6.9 | 15.7 | 40.5 | 29.9 | 21.7 | 71.0 | 453.9 | 76.1 | **1,078** | |  |
| PRR | 498.8 | 567.5 | 346.7 | 187.7 | 284.6 | 321.0 | 355.0 | 606.9 | 448.9 | 313.1 | 452.7 | 300.7 | 648.5 | 521.2 | 505.7 | 524.7 | 421.6 | 477.6 | 374.1 | 311.6 | 286.2 | **8,755** | |  |
| PODSTEM | 59.9 | 151.3 | 48.2 | 120.1 | 72.9 | 166.1 | 45.5 | 62.1 | 58.3 | 25.4 | 62.6 | 67.6 | 100.5 | 191.0 | 142.2 | 163.1 | 91.8 | 139.9 | 147.6 | 104.0 | 111.5 | **2,132** | |  |
| RAB | 21.4 | 15.5 | 42.2 | 4.8 | 1.1 | 7.2 | 7.3 | 10.6 | 9.4 | 3.1 | 3.3 | 3.1 | 4.8 | 0.8 | 37.8 | 40.3 | 23.3 | 6.8 | 31.4 | 7.0 | 23.1 | **304** | |  |
| RKNOT^d^ | 70.4 | 51.4 | 35.9 | 31.6 | 65.7 | 33.0 | 38.0 | 142.6 | 44.7 | 29.9 | 65.4 | 74.2 | 110.1 | 80.9 | 91.8 | 75.7 | 131.1 | 184.9 | 239.5 | 179.7 | 171.9 | **1,948** | |  |
| SSR | 325.5 | 368.2 | 158.0 | 16.5 | 60.3 | 14.0 | 21.6 | 22.1 | 507.5 | 42.0 | 109.5 | 64.0 | 143.5 | 722.2 | 320.1 | 212.0 | 68.6 | 258.7 | 450.3 | 393.9 | 384.8 | **4,663** | |  |
| SEED^e^ | 50.3 | 261.3 | 284.1 | 89.0 | 149.6 | 203.6 | 176.3 | 337.8 | 341.8 | 289.4 | 332.6 | 428.2 | 738.5 | 663.2 | 761.7 | 746.1 | 401.5 | 679.3 | 692.8 | 672.6 | 505.8 | **8,805** | |  |
| SEP | 37.6 | 46.0 | 48.6 | 19.4 | 60.4 | 17.1 | 21.7 | 30.3 | 212.2 | 46.3 | 172.0 | 140.2 | 293.3 | 293.9 | 377.3 | 314.7 | 127.4 | 358.9 | 325.4 | 269.8 | 190.7 | **3,403** | |  |
| SB | 2.7 | 2.5 | 2.9 | 5.1 | 2.2 | 1.2 | 1.4 | 2.6 | 2.4 | 1.7 | 1.7 | 2.2 | 2.8 | 3.0 | 3.5 | 9.6 | 15.3 | 1.5 | 8.2 | 4.0 | 4.4 | **81** | |  |
| SCN | 3,292.5 | 2,638.3 | 2,999.3 | 1,451.4 | 1,025.8 | 952.2 | 1,142.2 | 1,251.0 | 1,108.4 | 593.4 | 1,021.6 | 1,167.7 | 1,065.6 | 1,415.2 | 1,602.9 | 1,474.9 | 2,123.3 | 2,032.3 | 1,425.4 | 1,129.4 | 1,041.3 | **31,954** | |  |
| SR | 21.0 | 6.2 | 24.3 | 29.8 | 63.8 | 0.6 | 47.1 | 3.4 | 0.0 | 4.0 | 8.6 | 6.3 | 3.2 | 30.2 | 0.0 | 3.0 | 6.7 | 15.2 | 0.5 | 1.7 | 2.3 | **278** | |  |
| SC | 23.2 | 12.4 | 8.6 | 21.2 | 27.1 | 26.3 | 31.5 | 93.1 | 119.6 | 37.4 | 62.5 | 62.0 | 71.0 | 65.4 | 80.0 | 93.4 | 77.4 | 98.9 | 143.3 | 124.2 | 99.8 | **1,378** | |  |
| SDS | 45.0 | 115.5 | 303.3 | 923.1 | 567.4 | 147.6 | 233.8 | 138.7 | 349.8 | 161.3 | 230.3 | 277.8 | 250.7 | 414.5 | 949.4 | 367.3 | 386.7 | 432.5 | 693.4 | 414.7 | 474.2 | **7,877** | |  |
| VIRUS^f^ | 28.4 | 22.1 | 99.4 | 76.6 | 270.3 | 96.1 | 237.9 | 72.2 | 15.6 | 12.9 | 62.1 | 83.7 | 73.2 | 53.4 | 48.7 | 61.6 | 203.6 | 129.2 | 61.1 | 26.0 | 36.7 | **1,771** | |  |
| **Total** | **5,344** | **5,033** | **5,182** | **3,618** | **3,133** | **2,423** | **3,132** | **4,224** | **3,932** | **2,063** | **3,408** | **3,655** | **4,539** | **5,790** | **6,438** | **5,729** | **6,335** | **6,209** | **5,700** | **5,307** | **4,288** | **95,481** | |  |

^a^ Total values have been rounded to the nearest dollar amount and rounding errors may be present.

^b^ ANT = Anthracnose, BAC = Bacterial blight, BSR = Brown stem rot, CLBPSS = Cercospora leaf blight (purple seed stain), CHAR = Charcoal rot, DIAPHOM = Diaporthe-Phomopsis, DM = Downy mildew, FLS = Frogeye leaf spot, FW = Fusarium wilt, OTHER = Other diseases, PRR = Phytophthora root and stem rot, PODSTEM = Pod and stem blight, RAB = Rhizoctonia aerial blight, RKNOT = Root-knot and other nematodes, SSR = Sclerotinia stem rot (White mold), SEED = Seedling disease, SEP = Septoria brown spot, SB = Southern blight, SCN = Soybean cyst nematode, SR = Soybean rust, SC = Stem canker, SDS = Sudden death syndrome, VIRUS = Virus diseases.

^c^ Includes: black root rot, Cercospora leaf blight, *Cylindrocladium parasticum* (red crown rot), green stem syndrome, Neocosmospora root rot, Pythium root rot, target spot, and Texas root rot.

^d^ Includes: *Rotylenchulus reniformis* (reniform nematode), *Belonolaimus longicaudatus* (sting nematode), and *Meloidogyne* (root-knot nematodes), *Helicotylenchus* (spiral nematodes), *Hoplolaimus* (lance nematodes), *Paratrichodorus* (stubby root nematodes), and *Pratylenchus* spp. (lesion nematodes).

^e^ Includes: seedling diseases caused by a complex of organisms such as multiple species of *Fusarium*, *Pythium*, *Phomopsis*, and/or *Rhizoctonia solani*.

^f^ Includes: *Alfalfa mosaic virus*, *Bean pod mottle virus*, *Bean yellow mosaic virus*, *Peanut mottle virus*, *Soybean dwarf virus*, *Soybean mosaic virus*, *Soybean vein necrosis virus*, *Tobacco ringspot virus*, *Tobacco streak virus*, and *Tomato spotted wilt virus*.
