# Supplementary material for: Dissecting the economic impact of soybean diseases in the United States over two decades
Source: PLoS One. 2020 Apr 2;15(4):e0231141. doi: 10.1371/journal.pone.0231141 (PMC7117771; doi:10.1371/journal.pone.0231141)
Supplement: S5 Table — (DOCX) [file pone.0231141.s005.docx]

**Supplementary table 5.** Estimated cumulative soybean economic losses from 1996 to 2016 (in million USD) as a result of diseases affecting soybean from 12 states in the northern United States.

|  | **State (northern United States)^a^** | | | | | | | | | | | |  |
| --- | --- | --- | --- | --- | --- | --- | --- | --- | --- | --- | --- | --- | --- |
| **Disease** | **IA** | **IL** | **IN** | **KS** | **MI** | **MN** | **ND** | **NE** | **OH** | **PA** | **SD** | **WI** | **Total** |
| Anthracnose | 159.3 | 620.9 | 46.1 | 64.2 | 33.6 | 228.5 | 0.0 | 31.3 | 0.0 | 45.1 | 0.7 | 60.4 | **1,290** |
| Bacterial blight | 108.0 | 120.5 | 41.8 | 0.0 | 115.7 | 72.9 | 23.6 | 44.5 | 0.0 | 10.2 | 38.1 | 32.2 | **607** |
| Brown stem rot | 565.1 | 562.5 | 387.3 | 0.0 | 75.8 | 870.0 | 4.5 | 90.5 | 69.8 | 36.3 | 198.3 | 503.3 | **3,363** |
| Cercospora leaf blight (purple seed stain) | 219.0 | 239.9 | 77.7 | 15.8 | 85.2 | 46.6 | 9.0 | 40.7 | 165.4 | 55.0 | 44.3 | 94.4 | **1,093** |
| Charcoal rot | 524.0 | 1,341.1 | 202.9 | 1,460.4 | 268.3 | 171.7 | 87.9 | 67.7 | 408.6 | 35.1 | 278.0 | 364.4 | **5,210** |
| Diaporthe-Phomopsis | 52.0 | 271.6 | 83.8 | 6.6 | 66.1 | 32.5 | 0.0 | 19.2 | 42.0 | 97.0 | 57.1 | 109.2 | **837** |
| Downy mildew | 44.4 | 213.5 | 22.1 | 0.6 | 28.0 | 66.2 | 3.3 | 2.4 | 0.0 | 55.2 | 3.3 | 34.2 | **473** |
| Frogeye leaf spot | 177.5 | 274.3 | 112.5 | 4.7 | 6.8 | 28.2 | 0.0 | 25.5 | 120.3 | 29.1 | 1.1 | 35.7 | **816** |
| Fusarium wilt | 18.2 | 183.2 | 23.9 | 23.6 | 276.9 | 667.8 | 43.6 | 39.0 | 67.9 | 14.5 | 57.9 | 248.4 | **1,665** |
| Other diseases^b^ | 173.9 | 16.6 | 0.0 | 5.5 | 0.0 | 0.0 | 0.0 | 9.8 | 418.3 | 0.0 | 0.6 | 8.3 | **633** |
| Phytophthora root and stem rot | 402.3 | 944.9 | 1,204.1 | 82.2 | 234.3 | 815.9 | 408.3 | 93.3 | 2,909.7 | 27.0 | 651.1 | 349.7 | **8,123** |
| Pod and stem blight | 414.6 | 376.7 | 55.8 | 53.5 | 173.5 | 240.9 | 0.0 | 15.7 | 19.2 | 15.0 | 83.1 | 140.0 | **1,588** |
| Rhizoctonia aerial blight | 0.0 | 0.0 | 0.0 | 0.0 | 0.0 | 0.0 | 0.0 | 0.0 | 0.0 | 19.5 | 0.0 | 0.0 | **20** |
| Root-knot and other nematodes^c^ | 40.6 | 149.4 | 0.7 | 1.9 | 15.8 | 86.4 | 0.0 | 0.0 | 4.4 | 0.4 | 29.7 | 75.1 | **404** |
| Sclerotinia stem rot (White mold) | 970.9 | 855.3 | 219.3 | 0.2 | 581.8 | 533.4 | 69.7 | 78.6 | 471.2 | 105.8 | 191.5 | 583.0 | **4,661** |
| Seedling diseases^d^ | 697.6 | 871.7 | 421.6 | 1,136.8 | 497.1 | 899.7 | 994.0 | 206.0 | 1,440.5 | 58.0 | 503.6 | 330.2 | **8,057** |
| Septoria brown spot | 428.7 | 575.5 | 116.8 | 118.2 | 331.8 | 59.7 | 0.0 | 120.8 | 824.5 | 129.4 | 146.6 | 148.8 | **3,001** |
| Southern blight | 0.0 | 0.0 | 0.0 | 0.0 | 0.0 | 3.2 | 0.0 | 0.0 | 0.0 | 0.1 | 0.0 | 0.0 | **3** |
| Soybean cyst nematode | 11,520.6 | 4,755.0 | 1,681.8 | 428.4 | 1,328.3 | 3,093.6 | 240.4 | 609.8 | 2,918.5 | 1.5 | 1,474.5 | 871.7 | **28,924** |
| Soybean rust | 0.0 | 3.6 | 13.5 | 36.7 | 16.3 | 3.6 | 3.3 | 0.8 | 4.9 | 0.2 | 0.6 | 0.3 | **84** |
| Stem canker | 132.2 | 248.9 | 75.7 | 8.4 | 33.7 | 186.5 | 0.0 | 12.8 | 13.6 | 18.5 | 197.0 | 247.9 | **1,175** |
| Sudden death syndrome | 2,534.1 | 1,788.0 | 981.6 | 24.5 | 486.3 | 721.1 | 0.0 | 47.1 | 198.9 | 26.0 | 85.4 | 358.0 | **7,251** |
| Virus diseases^e^ | 696.6 | 261.6 | 60.2 | 14.8 | 64.1 | 0.0 | 0.4 | 89.2 | 142.7 | 28.3 | 92.4 | 163.6 | **1,614** |
| **Total** | **19,880** | **14,675** | **5,829** | **3,487** | **4,719** | **8,828** | **1,888** | **1,645** | **10,241** | **807** | **4,135** | **4,759** | **80,892** |

^a^ Total values have been rounded to the nearest dollar amount and rounding errors may be present.

^b^ Includes: black root rot, Cercospora leaf blight, *Cylindrocladium parasticum* (red crown rot), green stem syndrome, Neocosmospora root rot, Pythium root rot, target spot, and Texas root rot.

^c^ Includes: *Rotylenchulus reniformis* (reniform nematode), *Belonolaimus longicaudatus* (sting nematode), and *Meloidogyne* (root-knot nematodes), *Helicotylenchus* (spiral nematodes), *Hoplolaimus* (lance nematodes), *Paratrichodorus* (stubby root nematodes), and *Pratylenchus* spp. (lesion nematodes).

^d^ Includes: seedling diseases caused by a complex of organisms such as multiple species of *Fusarium*, *Pythium*, *Phomopsis*, and/or *Rhizoctonia solani*.

^e^ Includes: *Alfalfa mosaic virus*, *Bean pod mottle virus*, *Bean yellow mosaic virus*, *Peanut mottle virus*, *Soybean dwarf virus*, *Soybean mosaic virus*, *Soybean vein necrosis virus*, *Tobacco ringspot virus*, *Tobacco streak virus*, and *Tomato spotted wilt virus*.
