# Supplementary material for: Dissecting the economic impact of soybean diseases in the United States over two decades
Source: PLoS One. 2020 Apr 2;15(4):e0231141. doi: 10.1371/journal.pone.0231141 (PMC7117771; doi:10.1371/journal.pone.0231141)
Supplement: S6 Table — (DOCX) [file pone.0231141.s006.docx]

**Supplementary table 6.** Estimated cumulative soybean economic losses from 1996 to 2016 (in million USD) as a result of diseases affecting soybean from 16 states in the southern United States.

|  | **State (southern United States)^a^** | | | | | | | | | | | | | | | |  |
| --- | --- | --- | --- | --- | --- | --- | --- | --- | --- | --- | --- | --- | --- | --- | --- | --- | --- |
| **Disease** | **AL** | **AR** | **DE** | **FL** | **GA** | **KY** | **LA** | **MD** | **MO** | **MS** | **NC** | **OK** | **SC** | **TN** | **TX** | **VA** | **Total** |
| Anthracnose | 15.2 | 97.8 | 1.8 | 1.1 | 13.6 | 32.1 | 65.5 | 3.0 | 20.3 | 82.8 | 17.0 | 4.1 | 12.0 | 236.8 | 15.6 | 15.3 | **634** |
| Bacterial blight | 0.2 | 3.0 | 0.0 | 0.4 | 0.0 | 1.0 | 4.4 | 0.0 | 0.0 | 4.2 | 8.4 | 1.6 | 1.9 | 0.1 | 1.2 | 2.0 | **28** |
| Brown stem rot | 2.6 | 0.0 | 0.0 | 0.0 | 0.0 | 0.1 | 0.0 | 0.0 | 0.0 | 0.0 | 0.0 | 0.0 | 0.0 | 27.3 | 0.2 | 5.7 | **36** |
| Cercospora leaf blight (purple seed stain) | 21.9 | 42.5 | 0.5 | 0.4 | 2.3 | 9.6 | 223.7 | 4.7 | 58.6 | 180.7 | 19.3 | 4.9 | 7.3 | 90.2 | 13.5 | 13.6 | **694** |
| Charcoal rot | 26.1 | 702.3 | 10.5 | 1.8 | 4.2 | 224.2 | 112.0 | 6.9 | 275.0 | 577.6 | 13.9 | 36.5 | 4.6 | 253.2 | 21.1 | 4.2 | **2,274** |
| Diaporthe-Phomopsis | 17.8 | 62.3 | 1.8 | 1.4 | 9.6 | 94.0 | 51.8 | 1.2 | 20.8 | 181.2 | 48.3 | 7.4 | 15.2 | 206.1 | 7.8 | 8.0 | **735** |
| Downy mildew | 1.1 | 0.9 | 0.0 | 1.1 | 0.5 | 0.8 | 0.0 | 0.1 | 0.9 | 0.8 | 6.9 | 0.3 | 3.5 | 21.0 | 0.4 | 0.1 | **38** |
| Frogeye leaf spot | 14.4 | 82.2 | 5.1 | 2.0 | 4.7 | 34.1 | 87.6 | 14.4 | 122.6 | 248.7 | 39.7 | 2.7 | 14.2 | 434.3 | 12.0 | 26.2 | **1,145** |
| Fusarium wilt | 0.1 | 2.6 | 0.3 | 0.1 | 0.4 | 0.7 | 0.0 | 0.8 | 98.6 | 7.6 | 0.0 | 0.0 | 0.1 | 0.3 | 1.6 | 0.5 | **114** |
| Other diseases^b^ | 0.1 | 7.6 | 0.0 | 0.0 | 2.8 | 1.0 | 56.5 | 0.0 | 0.0 | 192.3 | 140.3 | 0.3 | 5.1 | 17.4 | 2.9 | 18.0 | **445** |
| Phytophthora root and stem rot | 0.0 | 5.6 | 0.0 | 0.0 | 0.0 | 17.6 | 19.1 | 0.2 | 500.5 | 45.8 | 32.9 | 2.2 | 0.2 | 5.6 | 2.0 | 0.3 | **632** |
| Pod and stem blight | 10.1 | 88.4 | 1.6 | 1.0 | 28.8 | 44.3 | 80.2 | 1.5 | 67.6 | 118.2 | 52.3 | 9.4 | 11.4 | 12.6 | 7.6 | 8.4 | **544** |
| Rhizoctonia aerial blight | 4.6 | 28.9 | 0.2 | 0.5 | 0.0 | 0.0 | 97.2 | 0.4 | 0.0 | 133.2 | 0.1 | 0.0 | 3.6 | 14.0 | 2.0 | 0.1 | **285** |
| Root-knot and other nematodes^c^ | 28.5 | 520.3 | 12.6 | 2.3 | 80.8 | 0.1 | 248.1 | 20.8 | 54.4 | 217.2 | 148.3 | 3.2 | 139.0 | 13.1 | 1.9 | 53.4 | **1,544** |
| Sclerotinia stem rot (White mold) | 0.0 | 0.0 | 0.0 | 0.0 | 0.0 | 0.0 | 0.0 | 0.1 | 0.0 | 1.8 | 0.0 | 0.0 | 0.0 | 0.1 | 0.5 | 0.0 | **3** |
| Seedling diseases^d^ | 13.3 | 91.1 | 1.9 | 1.9 | 5.1 | 56.3 | 22.0 | 0.9 | 186.8 | 115.2 | 17.6 | 9.0 | 2.1 | 197.7 | 3.7 | 24.0 | **749** |
| Septoria brown spot | 0.7 | 6.4 | 0.8 | 0.2 | 0.3 | 39.4 | 7.8 | 1.7 | 1.5 | 96.7 | 11.9 | 7.0 | 4.5 | 216.3 | 1.5 | 5.6 | **402** |
| Southern blight | 2.1 | 4.6 | 0.1 | 0.2 | 6.2 | 0.6 | 4.5 | 1.5 | 0.0 | 13.1 | 19.7 | 0.7 | 14.9 | 1.2 | 1.6 | 6.5 | **77** |
| Soybean cyst nematode | 11.9 | 407.3 | 30.1 | 0.8 | 14.1 | 330.0 | 19.0 | 56.2 | 1,135.6 | 68.5 | 400.1 | 30.6 | 51.9 | 380.8 | 0.1 | 93.1 | **3,030** |
| Soybean rust | 20.2 | 68.1 | 0.0 | 2.2 | 9.1 | 0.0 | 21.6 | 0.0 | 0.0 | 31.3 | 8.1 | 1.6 | 3.3 | 25.5 | 1.7 | 1.7 | **194** |
| Stem canker | 7.6 | 62.8 | 0.1 | 0.1 | 1.9 | 14.7 | 6.2 | 0.0 | 1.2 | 59.5 | 0.6 | 0.3 | 0.0 | 39.7 | 1.5 | 6.8 | **203** |
| Sudden death syndrome | 2.1 | 126.8 | 0.0 | 0.0 | 0.0 | 31.4 | 4.4 | 0.0 | 275.0 | 9.8 | 4.9 | 0.4 | 0.0 | 166.3 | 0.8 | 3.7 | **626** |
| Virus diseases^e^ | 5.6 | 6.0 | 1.4 | 0.1 | 0.3 | 14.1 | 8.2 | 4.2 | 3.5 | 63.1 | 25.0 | 0.6 | 20.5 | 0.6 | 1.5 | 2.3 | **157** |
| **Total** | **206** | **2,418** | **69** | **17** | **185** | **946** | **1,140** | **119** | **2,823** | **2,449** | **1,015** | **123** | **315** | **2,360** | **103** | **299** | **14,588** |

^a^ Total values have been rounded to the nearest dollar amount and rounding errors may be present.

^b^ Includes: black root rot, Cercospora leaf blight, *Cylindrocladium parasticum* (red crown rot), green stem syndrome, Neocosmospora root rot, Pythium root rot, target spot, and Texas root rot.

^c^ Includes: *Rotylenchulus reniformis* (reniform nematode), *Belonolaimus longicaudatus* (sting nematode), and *Meloidogyne* (root-knot nematodes), *Helicotylenchus* (spiral nematodes), *Hoplolaimus* (lance nematodes), *Paratrichodorus* (stubby root nematodes), and *Pratylenchus* spp. (lesion nematodes).

^d^ Includes: seedling diseases caused by a complex of organisms such as multiple species of *Fusarium*, *Pythium*, *Phomopsis*, and/or *Rhizoctonia solani*.

^e^ Includes: *Alfalfa mosaic virus*, *Bean pod mottle virus*, *Bean yellow mosaic virus*, *Peanut mottle virus*, *Soybean dwarf virus*, *Soybean mosaic virus*, *Soybean vein necrosis virus*, *Tobacco ringspot virus*, *Tobacco streak virus*, and *Tomato spotted wilt virus*.
