# Supplementary material for: Dissecting the economic impact of soybean diseases in the United States over two decades
Source: PLoS One. 2020 Apr 2;15(4):e0231141. doi: 10.1371/journal.pone.0231141 (PMC7117771; doi:10.1371/journal.pone.0231141)
Supplement: S8 Table — (DOCX) [file pone.0231141.s008.docx]

**Supplementary table 8.** Estimated cumulative soybean economic losses from 1996 to 2016 (in U.S. dollars per hectare) as a result of diseases affecting soybean from 16 states in the southern United States.

|  |  | **State (southern United States)^a^** | | | | | | | | | | | | | | |  |
| --- | --- | --- | --- | --- | --- | --- | --- | --- | --- | --- | --- | --- | --- | --- | --- | --- | --- |
| **Disease** | **AL** | **AR** | **DE** | **FL** | **GA** | **KY** | **LA** | **MD** | **MO** | **MS** | **NC** | **OK** | **SC** | **TN** | **TX** | **VA** | **Total** |
| Anthracnose | 121.2 | 75.2 | 23.4 | 99.4 | 102.5 | 62.6 | 171.9 | 15.9 | 10.1 | 120.7 | 27.9 | 30.2 | 62.1 | 457.4 | 146.7 | 71.3 | **1,598** |
| Bacterial blight | 3.9 | 2.3 | 0.1 | 35.8 | 0.0 | 2.1 | 7.9 | 0.0 | 0.0 | 5.3 | 13.6 | 12.0 | 10.7 | 0.2 | 10.3 | 9.4 | **114** |
| Brown stem rot | 18.3 | 0.0 | 0.1 | 3.1 | 0.0 | 0.1 | 0.0 | 0.1 | 0.0 | 0.0 | 0.0 | 0.0 | 0.0 | 53.5 | 1.8 | 25.1 | **102** |
| Cercospora leaf blight (purple seed stain) | 165.0 | 32.4 | 6.6 | 35.1 | 16.5 | 14.8 | 540.7 | 23.6 | 31.2 | 246.9 | 31.2 | 36.4 | 42.0 | 165.7 | 138.0 | 57.5 | **1,584** |
| Charcoal rot | 260.8 | 550.9 | 145.2 | 271.7 | 39.6 | 405.7 | 257.4 | 35.4 | 137.5 | 763.9 | 23.8 | 289.2 | 24.6 | 491.8 | 215.8 | 20.3 | **3,934** |
| Diaporthe-Phomopsis | 161.0 | 48.3 | 24.2 | 125.7 | 81.7 | 171.6 | 139.3 | 6.0 | 10.0 | 294.6 | 75.8 | 54.6 | 90.8 | 372.0 | 75.7 | 36.1 | **1,767** |
| Downy mildew | 9.7 | 0.7 | 0.2 | 105.7 | 5.9 | 1.5 | 0.0 | 0.6 | 0.4 | 1.1 | 11.8 | 1.7 | 19.5 | 41.2 | 4.1 | 0.5 | **205** |
| Frogeye leaf spot | 110.0 | 64.7 | 77.3 | 194.1 | 53.7 | 50.4 | 173.6 | 75.1 | 58.3 | 321.5 | 63.7 | 26.8 | 78.0 | 811.9 | 125.4 | 112.3 | **2,397** |
| Fusarium wilt | 0.9 | 2.0 | 3.9 | 10.0 | 3.3 | 1.4 | 0.0 | 4.2 | 44.9 | 9.6 | 0.0 | 0.0 | 0.3 | 0.4 | 14.8 | 2.0 | **98** |
| Other diseases^b^ | 1.5 | 6.1 | 0.4 | 0.0 | 25.3 | 1.4 | 109.8 | 0.0 | 0.0 | 235.2 | 245.9 | 2.2 | 25.7 | 27.8 | 48.0 | 79.1 | **808** |
| Phytophthora root and stem rot | 0.0 | 4.2 | 0.0 | 1.0 | 0.0 | 25.2 | 51.3 | 1.1 | 249.7 | 61.7 | 53.4 | 17.9 | 1.2 | 11.0 | 18.5 | 1.1 | **497** |
| Pod and stem blight | 78.7 | 67.7 | 22.7 | 98.1 | 222.4 | 77.6 | 205.3 | 7.9 | 31.3 | 186.2 | 84.1 | 71.8 | 57.5 | 22.7 | 74.1 | 37.1 | **1,345** |
| Rhizoctonia aerial blight | 35.2 | 22.4 | 3.6 | 44.4 | 0.0 | 0.0 | 234.3 | 1.8 | 0.0 | 169.9 | 0.1 | 0.0 | 24.6 | 27.5 | 25.1 | 0.3 | **589** |
| Root-knot and other nematodes^c^ | 268.0 | 408.5 | 178.5 | 211.5 | 753.9 | 0.1 | 566.0 | 105.6 | 24.7 | 254.0 | 247.3 | 21.5 | 765.1 | 26.6 | 18.7 | 237.8 | **4,088** |
| Sclerotinia stem rot (White mold) | 0.0 | 0.0 | 0.4 | 1.3 | 0.0 | 0.0 | 0.0 | 0.4 | 0.0 | 2.0 | 0.0 | 0.0 | 0.0 | 0.3 | 4.6 | 0.0 | **9** |
| Seedling diseases^d^ | 103.8 | 66.3 | 24.3 | 195.8 | 51.9 | 90.4 | 48.8 | 4.8 | 95.5 | 164.2 | 27.4 | 72.0 | 12.0 | 365.3 | 38.0 | 109.9 | **1,470** |
| Septoria brown spot | 5.7 | 5.0 | 10.0 | 18.6 | 4.5 | 66.7 | 15.7 | 9.3 | 0.7 | 124.2 | 19.7 | 51.6 | 25.3 | 397.6 | 14.4 | 25.2 | **794** |
| Southern blight | 16.9 | 3.7 | 0.8 | 11.5 | 55.6 | 1.2 | 8.4 | 9.1 | 0.0 | 16.4 | 31.1 | 5.2 | 82.7 | 2.6 | 17.2 | 29.5 | **292** |
| Soybean cyst nematode | 95.9 | 321.5 | 403.5 | 126.0 | 146.7 | 575.0 | 43.9 | 287.8 | 550.3 | 85.3 | 675.7 | 225.1 | 282.1 | 718.8 | 1.0 | 412.8 | **4,951** |
| Soybean rust | 175.4 | 52.7 | 0.0 | 185.9 | 108.8 | 0.0 | 72.0 | 0.0 | 0.0 | 38.7 | 14.8 | 17.5 | 19.7 | 51.9 | 33.4 | 7.5 | **778** |
| Stem canker | 65.2 | 49.3 | 1.2 | 10.8 | 21.9 | 24.0 | 12.5 | 0.1 | 0.6 | 84.7 | 1.0 | 1.9 | 0.0 | 77.8 | 13.0 | 27.2 | **391** |
| Sudden death syndrome | 17.6 | 97.8 | 0.3 | 20.4 | 0.0 | 52.9 | 8.1 | 0.3 | 126.4 | 14.8 | 7.1 | 2.8 | 0.3 | 307.6 | 7.6 | 14.5 | **678** |
| Virus diseases^e^ | 43.8 | 4.4 | 20.0 | 10.2 | 2.2 | 23.2 | 20.6 | 21.1 | 1.7 | 80.3 | 42.5 | 4.3 | 107.9 | 1.4 | 13.3 | 10.6 | **407** |
| **Total** | **1,758** | **1,886** | **947** | **1,816** | **1,696** | **1,648** | **2,687** | **610** | **1,373** | **3,281** | **1,698** | **945** | **1,732** | **4,433** | **1,059** | **1,327** | **28,896** |

^a^ Total values have been rounded to the nearest dollar amount and rounding errors may be present.

^b^ Includes: black root rot, Cercospora leaf blight, *Cylindrocladium parasticum* (red crown rot), green stem syndrome, Neocosmospora root rot, Pythium root rot, target spot, and Texas root rot.

^c^ Includes: *Rotylenchulus reniformis* (reniform nematode), *Belonolaimus longicaudatus* (sting nematode), and *Meloidogyne* (root-knot nematodes), *Helicotylenchus* (spiral nematodes), *Hoplolaimus* (lance nematodes), *Paratrichodorus* (stubby root nematodes), and *Pratylenchus* spp. (lesion nematodes).

^d^ Includes: seedling diseases caused by a complex of organisms such as multiple species of *Fusarium*, *Pythium*, *Phomopsis*, and/or *Rhizoctonia solani*.

^e^ Includes: *Alfalfa mosaic virus*, *Bean pod mottle virus*, *Bean yellow mosaic virus*, *Peanut mottle virus*, *Soybean dwarf virus*, *Soybean mosaic virus*, *Soybean vein necrosis virus*, *Tobacco ringspot virus*, *Tobacco streak virus*, and *Tomato spotted wilt virus*.
