# Supplementary material for: Dissecting the economic impact of soybean diseases in the United States over two decades
Source: PLoS One. 2020 Apr 2;15(4):e0231141. doi: 10.1371/journal.pone.0231141 (PMC7117771; doi:10.1371/journal.pone.0231141)
Supplement: S9 Table — (DOCX) [file pone.0231141.s009.docx]

**Supplementary table 9.** Estimated cumulative economic losses from 1996 to 2016 (in U.S. dollars per hectare) as a result of diseases affecting soybean from 12 states in the northern United States.

|  | **State (northern United States)^a^** | | | | | | | | | | | |  |
| --- | --- | --- | --- | --- | --- | --- | --- | --- | --- | --- | --- | --- | --- |
| **Disease** | **IA** | **IL** | **IN** | **KS** | **MI** | **MN** | **ND** | **NE** | **OH** | **PA** | **SD** | **WI** | **Total** |
| Anthracnose | 40.6 | 161.0 | 21.3 | 49.7 | 42.1 | 80.9 | 0.0 | 21.5 | 0.0 | 229.0 | 0.4 | 98.4 | **745** |
| Bacterial blight | 28.1 | 31.3 | 19.2 | 0.0 | 145.0 | 26.9 | 15.5 | 25.9 | 0.0 | 52.9 | 22.3 | 49.0 | **416** |
| Brown stem rot | 144.7 | 141.4 | 176.4 | 0.0 | 94.8 | 306.8 | 2.0 | 52.2 | 38.6 | 172.2 | 119.1 | 794.8 | **2,043** |
| Cercospora leaf blight (purple seed stain) | 55.8 | 61.7 | 35.8 | 12.6 | 106.3 | 15.3 | 3.9 | 22.0 | 86.3 | 296.6 | 26.1 | 142.9 | **865** |
| Charcoal rot | 125.9 | 349.1 | 94.3 | 1,175.1 | 338.3 | 63.3 | 49.9 | 36.0 | 221.7 | 189.1 | 163.7 | 537.6 | **3,344** |
| Diaporthe-Phomopsis | 12.7 | 69.6 | 38.7 | 7.3 | 82.1 | 11.8 | 0.0 | 10.9 | 23.0 | 578.3 | 33.0 | 165.2 | **1,032** |
| Downy mildew | 11.4 | 56.8 | 10.2 | 0.4 | 35.1 | 22.9 | 1.4 | 1.2 | 0.0 | 276.1 | 2.2 | 53.5 | **471** |
| Frogeye leaf spot | 45.8 | 73.7 | 51.6 | 3.1 | 8.6 | 9.8 | 0.0 | 12.9 | 66.7 | 143.0 | 0.5 | 54.0 | **470** |
| Fusarium wilt | 4.8 | 48.2 | 11.1 | 19.8 | 351.4 | 234.1 | 43.1 | 23.0 | 37.6 | 75.8 | 34.4 | 374.1 | **1,257** |
| Other diseases^b^ | 41.8 | 4.0 | 0.0 | 5.4 | 0.0 | 0.0 | 0.0 | 7.0 | 218.2 | 0.0 | 0.5 | 15.7 | **293** |
| Phytophthora root and stem rot | 98.5 | 238.2 | 549.2 | 68.5 | 295.1 | 289.6 | 322.1 | 49.4 | 1,596.6 | 135.3 | 390.4 | 522.0 | **4,555** |
| Pod and stem blight | 99.2 | 97.7 | 25.4 | 44.7 | 217.9 | 83.8 | 0.0 | 10.1 | 10.7 | 79.8 | 46.3 | 218.1 | **934** |
| Rhizoctonia aerial blight | 0.0 | 0.0 | 0.0 | 0.0 | 0.0 | 0.0 | 0.0 | 0.0 | 0.0 | 95.3 | 0.0 | 0.0 | **95** |
| Root-knot and other nematodes^c^ | 9.5 | 39.9 | 0.3 | 1.9 | 20.7 | 29.2 | 0.0 | 0.0 | 2.6 | 2.2 | 24.1 | 112.6 | **243** |
| Sclerotinia stem rot (white mold) | 242.9 | 219.9 | 100.3 | 0.2 | 718.7 | 187.8 | 43.1 | 51.8 | 255.8 | 624.1 | 109.0 | 879.4 | **3,433** |
| Seedling diseases^d^ | 175.8 | 225.3 | 191.2 | 856.0 | 622.4 | 314.4 | 627.7 | 108.5 | 779.2 | 290.9 | 303.1 | 516.6 | **5,011** |
| Septoria brown spot | 109.1 | 148.3 | 53.5 | 86.7 | 414.0 | 20.7 | 0.0 | 68.2 | 449.3 | 608.6 | 91.0 | 226.2 | **2,275** |
| Southern blight | 0.0 | 0.0 | 0.0 | 0.0 | 0.0 | 1.1 | 0.0 | 0.0 | 0.0 | 0.8 | 0.0 | 0.0 | **2** |
| Soybean cyst nematode | 2,828.4 | 1,223.0 | 768.7 | 322.0 | 1,680.4 | 1,101.5 | 112.0 | 310.7 | 1,584.2 | 9.5 | 852.0 | 1,313.3 | **12,106** |
| Soybean rust | 0.0 | 0.9 | 6.2 | 36.3 | 20.4 | 1.4 | 6.0 | 0.4 | 2.7 | 1.3 | 0.6 | 0.7 | **77** |
| Stem canker | 34.0 | 63.5 | 34.4 | 7.8 | 41.9 | 66.1 | 0.0 | 9.0 | 7.4 | 85.0 | 113.3 | 367.5 | **830** |
| Sudden death syndrome | 619.6 | 448.6 | 448.4 | 16.3 | 604.5 | 249.0 | 0.0 | 22.6 | 108.3 | 116.7 | 44.2 | 530.5 | **3,209** |
| Virus diseases^e^ | 171.2 | 67.9 | 27.6 | 13.4 | 80.4 | 0.0 | 0.4 | 52.4 | 79.3 | 137.8 | 54.3 | 249.5 | **934** |
| **Total** | **4,900** | **3,770** | **2,664** | **2,727** | **5,920** | **3,116** | **1,227** | **896** | **5,568** | **4,200** | **2,430** | **7,221** | **44,639** |

^a^Total values have been rounded to the near dollar amount and rounding errors may be present.

^b^ Includes: black root rot, Cercospora leaf blight, *Cylindrocladium parasticum* (red crown rot), green stem syndrome, Neocosmospora root rot, Pythium root rot, target spot, and Texas root rot.

^c^ Includes: *Rotylenchulus reniformis* (reniform nematode), *Belonolaimus longicaudatus* (sting nematode), and *Meloidogyne* (root-knot nematodes), *Helicotylenchus* (spiral nematodes), *Hoplolaimus* (lance nematodes), *Paratrichodorus* (stubby root nematodes), and *Pratylenchus* spp. (lesion nematodes).

^d^ Includes: seedling diseases caused by a complex of organisms such as multiple species of *Fusarium*, *Pythium*, *Phomopsis*, and/or *Rhizoctonia solani*.

^e^ Includes: *Alfalfa mosaic virus*, *Bean pod mottle virus*, *Bean yellow mosaic virus*, *Peanut mottle virus*, *Soybean dwarf virus*, *Soybean mosaic virus*, *Soybean vein necrosis virus*, *Tobacco ringspot virus*, *Tobacco streak virus*, and *Tomato spotted wilt virus*.
