# Supplementary material for: Dissecting the economic impact of soybean diseases in the United States over two decades
Source: PLoS One. 2020 Apr 2;15(4):e0231141. doi: 10.1371/journal.pone.0231141 (PMC7117771; doi:10.1371/journal.pone.0231141)
Supplement: S10 Table — (DOCX) [file pone.0231141.s010.docx]

**Supplementary table 10.** Estimated cumulative economic losses (in U.S. dollars per hectare) associated with soybean diseases (n=23) observed from a total of 28 states within each of two regions in the United States, pre- and post-discovery of soybean rust, harvest/yield/production zones (factors) of a period from 1996 to 2016.

| **Disease** | **Region** | | **Rust discovery^g^** | | **Harvest Zone^h^** | | | | **Yield Zone^i^** | | | | **Production Zone^f^** | | | | |
| --- | --- | --- | --- | --- | --- | --- | --- | --- | --- | --- | --- | --- | --- | --- | --- | --- | --- |
|  | **North^e^** | **South^f^** | **Post** | **Pre** | **HZ1** | **HZ2** | **HZ3** | **HZ4** | **YZ1** | **YZ2** | **YZ3** | **YZ4** | **PZ1** | **PZ2** | **PZ3** | **PZ4** |  |
| Anthracnose | 745 | 1,598 | 1,472 | 871 | 632 | 1,017 | 378 | 316 | 432 | 555 | 728 | 629 | 547 | 995 | 476 | 325 |  |
| Bacterial blight | 416 | 114 | 493 | 37 | 82 | 74 | 248 | 125 | 43 | 50 | 224 | 213 | 72 | 83 | 240 | 134 |  |
| Brown stem rot | 2,043 | 102 | 1,527 | 618 | 45 | 402 | 842 | 856 | 78 | 247 | 949 | 871 | 32 | 428 | 829 | 856 |  |
| Cercospora leaf blight (purple seed stain) | 865 | 1,584 | 1,814 | 635 | 578 | 1,024 | 533 | 313 | 403 | 532 | 691 | 822 | 435 | 1,033 | 662 | 318 |  |
| Charcoal rot | 3,344 | 3,934 | 4,762 | 2,516 | 1,273 | 1,331 | 3,525 | 1,149 | 2,077 | 1,695 | 2,364 | 1,142 | 1,236 | 1,634 | 3,368 | 1,039 |  |
| Diaporthe-Phomopsis | 1,032 | 1,767 | 1,714 | 1,086 | 1,053 | 1,012 | 551 | 183 | 831 | 611 | 687 | 671 | 859 | 1,062 | 696 | 183 |  |
| Downy mildew | 471 | 205 | 609 | 67 | 215 | 269 | 88 | 104 | 42 | 154 | 150 | 330 | 143 | 347 | 82 | 105 |  |
| Frogeye leaf spot | 470 | 2,397 | 2,259 | 607 | 652 | 1,319 | 591 | 304 | 315 | 568 | 913 | 1,070 | 596 | 1,103 | 847 | 319 |  |
| Fusarium wilt | 1,257 | 98 | 1,147 | 208 | 72 | 92 | 812 | 379 | 62 | 181 | 811 | 301 | 33 | 144 | 782 | 396 |  |
| Other diseases^a^ | 293 | 808 | 811 | 290 | 85 | 446 | 304 | 265 | 146 | 273 | 141 | 540 | 101 | 331 | 406 | 263 |  |
| Phytophthora root and stem rot | 4,555 | 497 | 3,382 | 1,670 | 86 | 295 | 1,942 | 2,730 | 349 | 877 | 1,750 | 2,076 | 41 | 388 | 1,830 | 2,794 |  |
| Pod and stem blight | 934 | 1,345 | 1,512 | 766 | 613 | 606 | 679 | 380 | 445 | 582 | 736 | 516 | 533 | 715 | 640 | 391 |  |
| Rhizoctonia aerial blight | 95 | 589 | 479 | 205 | 130 | 381 | 173 | 0 | 154 | 237 | 102 | 191 | 125 | 399 | 160 | 0 |  |
| Root-knot and other nematodes^b^ | 243 | 4,088 | 3,321 | 1,010 | 1,863 | 1,439 | 911 | 118 | 1,336 | 1,283 | 855 | 857 | 1,940 | 1,216 | 1,055 | 120 |  |
| Sclerotinia stem rot (white mold) | 3,433 | 9 | 2,658 | 784 | 385 | 364 | 1,664 | 1,028 | 38 | 291 | 1,248 | 1,864 | 239 | 546 | 1,614 | 1,043 |  |
| Seedling diseases^c^ | 5,011 | 1,470 | 5,119 | 1,363 | 564 | 881 | 3,009 | 2,028 | 752 | 1,483 | 1,809 | 2,438 | 480 | 950 | 3,055 | 1,998 |  |
| Septoria brown spot | 2,275 | 794 | 2,776 | 294 | 184 | 1,014 | 1,074 | 797 | 133 | 322 | 760 | 1,855 | 137 | 967 | 1,179 | 786 |  |
| Southern blight | 2 | 292 | 210 | 84 | 161 | 92 | 40 | 1 | 138 | 60 | 67 | 29 | 178 | 74 | 41 | 1 |  |
| Soybean cyst nematode | 12,106 | 4,951 | 10,751 | 6,306 | 1,124 | 2,463 | 4,949 | 8,521 | 1,686 | 2,875 | 4,708 | 7,787 | 1,274 | 2,410 | 4,843 | 8,530 |  |
| Soybean rust | 77 | 778 | 581 | 274 | 538 | 156 | 149 | 12 | 350 | 190 | 208 | 107 | 441 | 308 | 96 | 10 |  |
| Stem canker | 830 | 391 | 1,012 | 208 | 120 | 223 | 626 | 253 | 110 | 184 | 458 | 469 | 90 | 301 | 590 | 240 |  |
| Sudden death syndrome | 3,209 | 678 | 3,057 | 830 | 28 | 450 | 1,323 | 2,086 | 60 | 173 | 1,177 | 2,476 | 24 | 370 | 1,425 | 2,067 |  |
| Virus diseases^d^ | 934 | 407 | 792 | 549 | 153 | 332 | 464 | 393 | 226 | 122 | 407 | 587 | 183 | 319 | 446 | 394 |  |
| **Total** | **44,639** | **28,896** | **52,256** | **21,279** | **10,635** | **15,682** | **24,877** | **22,342** | **10,207** | **13,546** | **21,941** | **27,841** | **9,738** | **16,122** | **25,363** | **22,312** |  |
| ^a^ Includes: black root rot, Cercospora leaf blight, *Cylindrocladium parasticum* (red crown rot), green stem syndrome, Neocosmospora root rot, Pythium root rot, target spot, and Texas root rot.  ^b^ Includes: *Rotylenchulus reniformis* (reniform nematode), *Belonolaimus longicaudatus* (sting nematode), and *Meloidogyne* (root-knot nematodes), *Helicotylenchus* (spiral nematodes), *Hoplolaimus* (lance nematodes), *Paratrichodorus* (stubby root nematodes), and *Pratylenchus* spp. (lesion nematodes).  ^c^ Includes: seedling diseases caused by a complex of organisms such as multiple species of *Fusarium*, *Pythium*, *Phomopsis*, and/or *Rhizoctonia solani*.  ^d^ Includes: *Alfalfa mosaic virus*, *Bean pod mottle virus*, *Bean yellow mosaic virus*, *Peanut mottle virus*, *Soybean dwarf virus*, *Soybean mosaic virus*, *Soybean vein necrosis virus*, *Tobacco ringspot virus*, *Tobacco streak virus*, and *Tomato spotted wilt virus*.  ^e^ Includes: Alabama, Arkansas, Delaware, Florida, Georgia, Kentucky, Louisiana, Maryland, Mississippi, Missouri, North Carolina, Oklahoma, South Carolina, Tennessee, Texas, and Virginia.  ^f^ Includes: Illinois, Indiana, Iowa, Kansas, Michigan, Minnesota, Nebraska, North Dakota, Ohio, Pennsylvania, South Dakota, and Wisconsin. Total values have been rounded to the nearest dollar amount and rounding errors may be present.  ^g^ For the purpose of this study, post-discovery of soybean rust spans from 2004 to 2016 while pre-discovery spans from 1996 to 2003.  ^k,l,m^ Represent four levels (zone 1-4) based on the quartiles within a data base containing 588 yield (kg/ha)/harvest area (ha)/production (MT) data points (588 = 21 years × 28 states). The data points within the minimum to first quartile are classified as zone 1. Similarly, data points from the first quartile to median, median to third quartile, and > third quartile were respectively classified as zones 2, 3, and 4. | | | | | | | | | | | | | | | | | |
