# Supplementary material for: Dissecting the economic impact of soybean diseases in the United States over two decades
Source: PLoS One. 2020 Apr 2;15(4):e0231141. doi: 10.1371/journal.pone.0231141 (PMC7117771; doi:10.1371/journal.pone.0231141)
Supplement: S11 Table — (DOCX) [file pone.0231141.s011.docx]

**Supplementary table 11.** Estimated cumulative soybean economic losses (in U.S. dollars per hectare) due to disease categories across 28 states within the United States (AL, AR, DE, FL, GA, IA, IL, IN, KS, KY, LA, MD, MI, MN, MO, NC, ND, NE, OH, OK, PA, SC, SD, TN, TX, VA, and WI) from 1996 to 2016.

| **Category** | Year^a^ | | | | | | | | | | | | | | | | | | | | | |
| --- | --- | --- | --- | --- | --- | --- | --- | --- | --- | --- | --- | --- | --- | --- | --- | --- | --- | --- | --- | --- | --- | --- |
|  | **1996** | **1997** | **1998** | **1999** | **2000** | **2001** | **2002** | **2003** | **2004** | **2005** | **2006** | **2007** | **2008** | **2009** | **2010** | **2011** | **2012** | **2013** | **2014** | **2015** | **2016** | **Total** |
| Bacterial^b^ | 11 | 7 | 4 | 2 | 2 | 2 | 5 | 5 | 12 | 10 | 12 | 39 | 49 | 47 | 69 | 121 | 49 | 17 | 20 | 17 | 32 | **530** |
| Foliar^c^ | 566 | 503 | 518 | 325 | 340 | 523 | 1,145 | 886 | 956 | 572 | 775 | 673 | 971 | 1,667 | 969 | 1,278 | 1,061 | 1,363 | 1,074 | 978 | 878 | **18,021** |
| Nematode^d^ | 1,470 | 1,162 | 1,160 | 691 | 584 | 620 | 687 | 942 | 716 | 500 | 759 | 900 | 1,023 | 1,077 | 1,179 | 1,215 | 1,838 | 1,620 | 1,287 | 969 | 989 | **21,388** |
| Stem/Root^e^ | 1,159 | 1,255 | 1,149 | 915 | 843 | 682 | 905 | 1,374 | 1,161 | 770 | 1,100 | 1,258 | 1,670 | 1,840 | 2,219 | 2,783 | 3,294 | 2,035 | 1,945 | 1,502 | 1,296 | **31,154** |
| Virus^f^ | 57 | 32 | 53 | 42 | 133 | 55 | 117 | 62 | 30 | 15 | 32 | 42 | 35 | 33 | 35 | 109 | 180 | 108 | 76 | 51 | 47 | **1,342** |
| Other^g^ | 29 | 74 | 9 | 10 | 49 | 33 | 38 | 48 | 56 | 13 | 11 | 26 | 18 | 19 | 26 | 62 | 64 | 39 | 97 | 266 | 114 | **1,101** |
| **Total** | **3,291** | **3,032** | **2,892** | **1,984** | **1,951** | **1,914** | **2,897** | **3,317** | **2,930** | **1,881** | **2,688** | **2,938** | **3,767** | **4,682** | **4,497** | **5,568** | **6,486** | **5,182** | **4,499** | **3,781** | **3,356** | **73,535** |

^a^ Total values have been rounded to the nearest dollar amount and rounding errors may be present.

^b^ Includes: Bacterial blight.

^c^ Includes: Anthracnose, Cercospora leaf blight (purple seed stain), Diaporthe-Phomopsis, Downy mildew, Frogeye leaf spot, Pod and stem blight, Rhizoctonia aerial blight, Septoria leaf spot, and Soybean rust

^d^ Includes: *Heterodera glycine* (soybean cyst nematode), *Meloidogyne* spp. (root-knot nematodes), *Rotylenchulus reniformis* (reniform nematode), *Belonolaimus longicaudatus* (sting nematode), *Helicotylenchus* (spiral nematodes), *Hoplolaimus* (lance nematodes), *Paratrichodorus* (stubby root nematodes), and *Pratylenchus* spp. (lesion nematodes).

^e^ Includes: Brown stem rot, Charcoal rot, Fusarium wilt, Phytophthora root and stem rot, Sclerotinia stem rot (white mold), Seedling diseases (caused by a complex of organisms such as multiple species of *Fusarium*, *Pythium*, *Phomopsis*, and/or *Rhizoctonia solani*), Southern blight, Stem canker, and Sudden death syndrome.

^f^ Includes: *Alfalfa mosaic virus*, *Bean pod mottle virus*, *Bean yellow mosaic virus*, *Peanut mottle virus*, *Soybean dwarf virus*, *Soybean mosaic virus*, *Soybean vein necrosis virus*, *Tobacco ringspot virus*, *Tobacco streak virus*, and *Tomato spotted wilt virus*.

^g^ Includes: black root rot, Cercospora leaf blight, *Cylindrocladium parasticum* (red crown rot), green stem syndrome, Neocosmospora root rot, Pythium root rot, target spot, and Texas root rot.
