# Supplementary material for: Dissecting the economic impact of soybean diseases in the United States over two decades
Source: PLoS One. 2020 Apr 2;15(4):e0231141. doi: 10.1371/journal.pone.0231141 (PMC7117771; doi:10.1371/journal.pone.0231141)
Supplement: S12 Table — (DOCX) [file pone.0231141.s012.docx]

**Supplementary table 12.** Estimated cumulative soybean economic losses from 1996 to 2016 (in U.S. dollars per hectare) due to disease categories in 16 southern and 12 northern states within the United States.

| Region | State | Category^a^ | | | | | | |  | |
| --- | --- | --- | --- | --- | --- | --- | --- | --- | --- | --- |
|  |  | Bacterial^b^ | Foliar^c^ | Nematode^d^ | Stem/Root^e^ | Virus^f^ | Other^g^ | **Total** | |  |
| South | AL | 3.9 | 861.8 | 363.9 | 483.4 | 43.8 | 1.5 | **1,758** | |  |
|  | AR | 2.3 | 369.1 | 730.0 | 774.1 | 4.4 | 6.1 | **1,886** | |  |
|  | DE | 0.1 | 168.0 | 582.0 | 176.3 | 20.0 | 0.4 | **947** | |  |
|  | FL | 35.8 | 907.0 | 337.5 | 525.6 | 10.2 | 0.0 | **1,816** | |  |
|  | GA | 0.0 | 596.1 | 900.6 | 172.3 | 2.2 | 25.3 | **1,696** | |  |
|  | KY | 2.1 | 445.1 | 575.1 | 600.9 | 23.2 | 1.4 | **1,648** | |  |
|  | LA | 7.9 | 1552.8 | 609.9 | 386.5 | 20.6 | 109.8 | **2,687** | |  |
|  | MD | 0.0 | 140.2 | 393.3 | 55.3 | 21.1 | 0.0 | **610** | |  |
|  | MO | 0.0 | 142.0 | 575.0 | 654.5 | 1.7 | 0.0 | **1,373** | |  |
|  | MS | 5.3 | 1503.8 | 339.3 | 1117.3 | 80.3 | 235.2 | **3,281** | |  |
|  | NC | 13.6 | 329.1 | 922.9 | 143.7 | 42.5 | 245.9 | **1,698** | |  |
|  | OK | 12.0 | 290.6 | 246.6 | 389.0 | 4.3 | 2.2 | **945** | |  |
|  | SC | 10.7 | 419.3 | 1047.1 | 121.1 | 107.9 | 25.7 | **1,732** | |  |
|  | TN | 0.2 | 2347.8 | 745.4 | 1310.2 | 1.4 | 27.8 | **4,433** | |  |
|  | TX | 10.3 | 636.8 | 19.7 | 331.1 | 13.3 | 48.0 | **1,059** | |  |
|  | VA | 9.4 | 347.6 | 650.6 | 229.6 | 10.6 | 79.1 | **1,327** | |  |
|  | **Total** | **114** | **11,057** | **9,039** | **7,471** | **407** | **808** | **28,896** | |  |
| North | IA | 28.1 | 374.5 | 2837.9 | 1446.2 | 171.2 | 41.8 | **4,900** | |  |
|  | IL | 31.3 | 669.6 | 1262.9 | 1734.0 | 67.9 | 4.0 | **3,770** | |  |
|  | IN | 19.2 | 242.7 | 769.0 | 1605.2 | 27.6 | 0.0 | **2,664** | |  |
|  | KS | 0.0 | 240.9 | 323.9 | 2143.7 | 13.4 | 5.4 | **2,727** | |  |
|  | MI | 145.0 | 926.3 | 1701.2 | 3067.0 | 80.4 | 0.0 | **5,920** | |  |
|  | MN | 26.9 | 246.5 | 1130.7 | 1712.3 | 0.0 | 0.0 | **3,116** | |  |
|  | ND | 15.5 | 11.3 | 112.0 | 1087.9 | 0.4 | 0.0 | **1,227** | |  |
|  | NE | 25.9 | 147.1 | 310.7 | 352.5 | 52.4 | 7.0 | **896** | |  |
|  | OH | 0.0 | 638.5 | 1586.8 | 3045.1 | 79.3 | 218.2 | **5,568** | |  |
|  | PA | 52.9 | 2307.8 | 11.7 | 1689.8 | 137.8 | 0.0 | **4,200** | |  |
|  | SD | 22.3 | 200.0 | 876.1 | 1277.1 | 54.3 | 0.5 | **2,430** | |  |
|  | WI | 49.0 | 958.8 | 1425.9 | 4522.5 | 249.5 | 15.7 | **7,221** | |  |
|  | **Total** | **416** | **6,964** | **12,349** | **23,683** | **934** | **293** | **44,639** | |  |

^a^ Total values have been rounded to the nearest dollar amount and rounding errors may be present.

^b^ Includes: Bacterial blight.

^c^ Includes: Anthracnose, Cercospora leaf blight (purple seed stain), Diaporthe-Phomopsis, Downy mildew, Frogeye leaf spot, Pod and stem blight, Rhizoctonia aerial blight, Septoria leaf spot, and Soybean rust

^d^ Includes: *Heterodera glycine* (soybean cyst nematode), *Meloidogyne* spp. (root-knot nematodes), *Rotylenchulus reniformis* (reniform nematode), *Belonolaimus longicaudatus* (sting nematode), *Helicotylenchus* (spiral nematodes), *Hoplolaimus* (lance nematodes), *Paratrichodorus* (stubby root nematodes), and *Pratylenchus* spp. (lesion nematodes).

^e^ Includes: Brown stem rot, Charcoal rot, Fusarium wilt, Phytophthora root and stem rot, Sclerotinia stem rot (white mold), Seedling diseases (caused by a complex of organisms such as multiple species of *Fusarium*, *Pythium*, *Phomopsis*, and/or *Rhizoctonia solani*), Southern blight, Stem canker, and Sudden death syndrome.

^f^ Includes: *Alfalfa mosaic virus*, *Bean pod mottle virus*, *Bean yellow mosaic virus*, *Peanut mottle virus*, *Soybean dwarf virus*, *Soybean mosaic virus*, *Soybean vein necrosis virus*, *Tobacco ringspot virus*, *Tobacco streak virus*, and *Tomato spotted wilt virus*.

^g^ Includes: black root rot, Cercospora leaf blight, *Cylindrocladium parasticum* (red crown rot), green stem syndrome, Neocosmospora root rot, Pythium root rot, target spot, and Texas root rot.
