# Supplementary material for: Dissecting the economic impact of soybean diseases in the United States over two decades
Source: PLoS One. 2020 Apr 2;15(4):e0231141. doi: 10.1371/journal.pone.0231141 (PMC7117771; doi:10.1371/journal.pone.0231141)
Supplement: S13 Table — (DOCX) [file pone.0231141.s013.docx]

**Supplementary table 13.** Estimated cumulative economic losses (from 1996 to 2016 in U.S. dollars per hectare) due to disease categories observed from a total of 28 states within each of two regions in the United States, pre- and post-discovery of soybean rust, harvest/yield/production zones.

| Category | Region | | Rust discovery^j^ | | Harvest Zone^k^ | | | | Yield Zone^l^ | | | | Production Zone^m^ | | | |
| --- | --- | --- | --- | --- | --- | --- | --- | --- | --- | --- | --- | --- | --- | --- | --- | --- |
|  | North^h^ | South^i^ | Post | Pre | HZ1 | HZ2 | HZ3 | HZ4 | YZ1 | YZ2 | YZ3 | YZ4 | PZ1 | PZ2 | PZ3 | PZ4 |
| Bacterial^a^ | 416.0 | 113.5 | 492.9 | 36.7 | 81.9 | 74.0 | 248.4 | 125.3 | 43.0 | 49.6 | 224.0 | 213.0 | 72.4 | 82.9 | 240.3 | 134.0 |
| Foliar^b^ | 6,964.0 | 11,057.2 | 13,215.2 | 4,806.0 | 4,593.5 | 6,798.9 | 4,217.1 | 2,411.7 | 3,105.5 | 3,750.3 | 4,974.7 | 6,190.7 | 3,815.4 | 6,929.1 | 4,839.6 | 2,437.1 |
| Nematode^c^ | 12,348.6 | 9,039.0 | 14,072.2 | 7,315.4 | 2,987.6 | 3,902.4 | 5,859.2 | 8,638.5 | 3,022.3 | 4,158.0 | 5,562.6 | 8,644.8 | 3,213.5 | 3,626.1 | 5,898.0 | 8,650.1 |
| Stem/Root^d^ | 23,683.4 | 7,471.1 | 22,873.0 | 8,281.4 | 2,733.9 | 4,128.9 | 13,783.5 | 10,508.1 | 3,664.0 | 5,192.8 | 10,631.9 | 11,665.8 | 2,352.3 | 4,834.6 | 13,532.9 | 10,434.6 |
| Virus^e^ | 934.2 | 407.3 | 792.2 | 549.3 | 152.8 | 331.5 | 464.2 | 393.1 | 226.1 | 121.9 | 406.7 | 586.8 | 182.9 | 318.6 | 446.1 | 393.9 |
| Other^f^ | 292.6 | 808.3 | 810.9 | 290.0 | 85.4 | 446.1 | 304.2 | 265.3 | 145.9 | 273.5 | 141.2 | 540.3 | 101.4 | 331.1 | 405.9 | 262.5 |
| **Total^g^** | **44,639** | **28,896** | **52,256** | **21,279** | **10,635** | **15,682** | **24,877** | **22,342** | **10,207** | **13,546** | **21,941** | **27,841** | **9,738** | **16,122** | **25,363** | **22,312** |

^a^ Includes: Bacterial blight.

^b^ Includes: Anthracnose, Cercospora leaf blight (purple seed stain), Diaporthe-Phomopsis, Downy mildew, Frogeye leaf spot, Pod and stem blight, Rhizoctonia aerial blight, Septoria leaf spot, and Soybean rust

^c^ Includes: *Heterodera glycine* (soybean cyst nematode), *Meloidogyne* spp. (root-knot nematodes), *Rotylenchulus reniformis* (reniform nematode), *Belonolaimus longicaudatus* (sting nematode), *Helicotylenchus* (spiral nematodes), *Hoplolaimus* (lance nematodes), *Paratrichodorus* (stubby root nematodes), and *Pratylenchus* spp. (lesion nematodes).

^d^ Includes: Brown stem rot, Charcoal rot, Fusarium wilt, Phytophthora root and stem rot, Sclerotinia stem rot (white mold), Seedling diseases (caused by a complex of organisms such as multiple species of *Fusarium*, *Pythium*, *Phomopsis*, and/or *Rhizoctonia solani*), Southern blight, Stem canker, and Sudden death syndrome.

^e^ Includes: *Alfalfa mosaic virus*, *Bean pod mottle virus*, *Bean yellow mosaic virus*, *Peanut mottle virus*, *Soybean dwarf virus*, *Soybean mosaic virus*, *Soybean vein necrosis virus*, *Tobacco ringspot virus*, *Tobacco streak virus*, and *Tomato spotted wilt virus*.

^f^ Includes: black root rot, Cercospora leaf blight, *Cylindrocladium parasticum* (red crown rot), green stem syndrome, Neocosmospora root rot, Pythium root rot, target spot, and Texas root rot.

^g^ Total values have been rounded to the nearest dollar amount and rounding errors may be present.

^h^ Includes: Illinois, Indiana, Iowa, Kansas, Michigan, Minnesota, Nebraska, North Dakota, Ohio, Pennsylvania, South Dakota, and Wisconsin.

^i^ Includes: Alabama, Arkansas, Delaware, Florida, Georgia, Kentucky, Louisiana, Maryland, Mississippi, Missouri, North Carolina, Oklahoma, South Carolina, Tennessee, Texas, and Virginia.

^j^ For the purpose of this study, period of post-discovery of soybean rust spans from 2004 to 2016 while pre-discovery spans from 1996 to 2003.

^k, l, m^ Represent four levels (zone 1-4) based on the quartiles within a data base containing 588 yield (kg/ha)/harvest area (ha)/production (MT) data points (588 = 21 years × 28 states). The data points within the minimum to first quartile are classified as zone 1. Similarly, data points from the first quartile to median, median to third quartile, and > third quartile were respectively classified as zones 2, 3, and 4.
